# Integrated analysis of mRNA and long noncoding RNA profiles in peripheral blood mononuclear cells of patients with bronchial asthma

**DOI:** 10.1186/s12890-022-01945-9

**Published:** 2022-05-02

**Authors:** Han Cui, Ruirui Duan, Hongtao Niu, Tao Yu, Ke Huang, Chen Chen, Ke Hao, Ting Yang, Chen Wang

**Affiliations:** 1 Department of Pathophysiology, State Key Laboratory of Medical Molecular Biology, Institute of Basic Medical Sciences, Peking Union Medical College, Chinese Academy of Medical Sciences, Beijing, China; 2 Peking University China-Japan Friendship School of Clinical Medicine, Beijing, China; 3 Department of Pulmonary and Critical Care Medicine, China-Japan Friendship Hospital, Beijing, China; 4 Institute of Respiratory Medicine, Peking Union Medical College, Chinese Academy of Medical Science, Beijing, China; 5 Department of Genetics and Genomic Sciences, Icahn School of Medicine at Mount Sinai, New York, NY USA; 6 Department of Geriatric, Beijing Hospital, Beijing, China

**Keywords:** Bronchial asthma, Long non-coding RNA, mRNA, Peripheral blood mononuclear cell, Microarray analysis

## Abstract

**Background:**

Bronchial asthma is a heterogeneous disease with distinct disease phenotypes and underlying pathophysiological mechanisms. Long non-coding RNAs (lncRNAs) are involved in numerous functionally different biological and physiological processes. The aim of this study was to identify differentially expressed lncRNAs and mRNAs in patients with asthma and further explore the functions and interactions between lncRNAs and mRNAs.

**Methods:**

Ten patients with asthma and 9 healthy controls were enrolled in this study. RNA was isolated from peripheral blood mononuclear cells. We performed microarray analysis to evaluate lncRNA and mRNA expression. The functions of the differentially expressed mRNAs were analyzed by Gene Ontology and Kyoto Encyclopedia of Genes and Genomes pathway analyses. A global signal transduction network was constructed to identify the core mRNAs. An lncRNA–mRNA network was constructed. Five mRNAs showing the greatest differences in expression levels or high degrees in the gene–gene functional interaction network, with their correlated lncRNAs, were validated by real-time quantitative polymerase chain reaction.

**Results:**

We identified 2229 differentially expressed mRNAs and 1397 lncRNAs between the asthma and control groups. Kyoto Encyclopedia of Genes and Genomes pathway analysis identified many pathways associated with inflammation and cell survival. The gene–gene functional interaction network suggested that some core mRNAs are involved in the pathogenesis of bronchial asthma. The lncRNA–mRNA co-expression network revealed correlated lncRNAs. *CXCL8*, *FOXO3*, *JUN*, *PIK3CA*, and *G0S2* and their related lncRNAs NONHSAT115963, AC019050.1, MTCYBP3, KB-67B5.12, and HNRNPA1P12 were identified according to their differential expression levels and high degrees in the gene–gene network.

**Conclusions:**

We identified the core mRNAs and their related lncRNAs and predicted the biological processes and signaling pathways involved in asthma.

**Supplementary Information:**

The online version contains supplementary material available at 10.1186/s12890-022-01945-9.

## Introduction

Bronchial asthma is a heterogeneous disease characterized by chronic airway inflammation, reversible airway obstruction, and bronchial hypersensitivity [1]. At present, the number of people suffering from asthma worldwide has reached more than 300 million, making asthma one of the most common chronic lung diseases [2]. Asthma has long been considered as a T helper 2-cell-mediated disease; furthermore, basophils, type 2 innate lymphoid cells, and mast cells can produce T helper 2-cell-associated cytokines [3]. It has been suggested that asthma is a highly heterogeneous disease with complexed pathophysiological mechanisms [4]. Omics technologies may help to reveal the complex mechanisms underlying the pathophysiology of asthma.

Long noncoding RNA (lncRNA) is a type of noncoding RNA composed of more than 200 nucleotides [5]. Initially, lncRNAs were regarded as ‘noise’ in the process of genomic transcription, and were assumed to not have biological functions. However, an increasing number of studies have indicated that lncRNAs are closely related to many physiological activities and pathological processes, such as epigenetic modification, cell differentiation, and development and transcriptional regulation [5]. The largest public dataset from the GENCODE project provides information on 15,877 human lncRNA genes (http://www.gencodegenes.org/stats/archive.html#a21). Based on these data, several studies have shown that lncRNAs are involved in a variety of biological and physiological processes and are directly related to various cancers, coronary artery disease, and many other diseases affecting humans [6–8]. It is reported that LncRNAs also play many regulatory roles in the pathological process of asthma, including helper T cell (Th)1/Th2 imbalance, eosinophil dysfunction, macrophage polarization, airway smooth muscle cell proliferation and glucocorticoid insensitivity [9]. Technological advances have allowed for a better understanding of the complex pathogenesis of asthma. To provide clues regarding the crucial pathways and pivotal pathogenic genes involved in the pathogenesis of asthma, we identified the lncRNAs and mRNAs in peripheral blood mononuclear cells (PBMCs) from patients with bronchial asthma. Furthermore, we comprehensively analyzed the function and interactions of lncRNA and mRNA.

## Materials and methods

### Study population

Ten patients with bronchial asthma and 9 healthy controls participated in the study conducted at China-Japan Friendship Hospital (Beijing, China) between July 2017 and December 2018. They were diagnosed as bronchial asthma according to the guidelines of Global Initiative for Asthma (history of asthma symptoms and record of reversible airflow limitation and a forced expiratory volume in the first second (FEV1) reversibility ≥ 12% and 200 mL after post-bronchodilator spirometry) [10]. The inclusion criteria for the patients were: (1) age ≥ 18 years; (2) medical reports of treating physicians, symptoms, and use of medications for asthma were available and (3) receiving regular treatment of anti-asthma medication with no active asthma nor exacerbation. Nine age- and sex- matched controls from the general population who underwent physical examinations at the China-Japan Friendship Hospital were recruited for this study; these subjects had (1) no previous or present diagnosis of chronic obstructive pulmonary disease, asthma, or other respiratory diseases; (2) no history of allergic diseases, including skin symptoms and nasal symptoms; (3) no history of chronic cough, wheezing, shortness of breath, or expectoration; 4) no history of other system diseases, such as digestive diseases or autoimmune diseases. Patients and controls were excluded based on the following criteria: (1) pregnancy or breast feeding; (2) liver and kidney insufficiency; (3) past or present malignant tumors; and (4) diagnosis of acute infectious disease. All participants signed a written informed consent form. Furthermore, the study was approved by the Ethics Committee of the China-Japan Friendship Hospital (2017–19). The study was carried out in accordance with the Declaration of Helsinki.

### PBMC isolation and RNA extraction

PBMCs were isolated from the peripheral blood as previously reported [11]. We collected venous blood (6 mL) from each participant using an EDTA-anticoagulated tube. PBMCs were isolated via density gradient centrifugation using Ficoll-Paque PLUS (GE Healthcare, Little Chalfont, UK) and stored at − 80 °C. Total RNA was isolated using TRIzol reagent (Life Technologies, Carlsbad, CA, USA) and purified with an RNeasy Mini Kit (Qiagen, Hilden, Germany). RNA was quantitatively detected using a spectrophotometer (NanoDrop 1000, Thermo Fisher Scientific, Waltham, MA, USA; 260 nm absorbance).

### Microarray profiling and data analysis

The Cnkingbio Biotechnology Corporation (Beijing, China) provided protocols for assessing the expression profiles using microarray analysis. Biotinylated cDNA was prepared according to the standard Affymetrix protocol using 250 ng of total RNA with an Ambion® WT Expression Kit (Austin, TX, USA). The labeled cDNA was hybridized at 45 °C for 16 h using the genechip (Clariom D Assay, human, Thermo Fisher Scientific). An Affymetrix Jet Station 450 (Santa Clara, CA, USA) was used to stain and clean genes. All arrays were scanned using the Affymetrix GeneChip Command Console software, which was installed on the GeneChip Scanner 3000 7G.

The ‘limma’ R package (version 3.36.5) was used to filter the differentially expressed genes (DEGs) identified by microarray analysis. Concurrently, for statistical analysis, Student’s *t*-test was used to screen genes with differential expression. The Benjamini–Hochberg method was used to correct for multiple tests, and the false discovery rate was used to adjust the *P*-values for multiple comparisons [12]. The cutoff criteria of differentially expressed mRNAs was fold-change (|FC|) > 1.5 and *P* < 0.05. Hierarchical clustering was performed based on differentially expressed mRNAs using the R package ‘pheatmap’ (version: 1.0.12).

### GO analysis and KEGG pathway analysis

Gene Ontology (GO) analysis was performed to determine the primary functions of the differentially expressed mRNAs. Kyoto Encyclopedia of Genes and Genomes (KEGG) is a knowledge databank for systematic analysis of gene functions and significantly altered pathways, which links genomic information with higher-order functional information [13–15]. Two-sided Fisher's exact test was used as the statistical analysis method, and the Benjamini–Hochberg method was used for multiple test correction. The analysis product of Cnkingbio Biotechnology Corporation was used, and the internal name is GO_Enrichment_Analysis/Pathway_Enrichment_Analysis. The threshold set for the significantly changed pathways was *P* < 0.05.

### Global signal transduction network

A global signal transduction network (signal-net) was constructed to identify interactions between DEGs in patients with asthma. We used Cytoscape software (version 3.6.0) for network visualization. In the network graph, edges and nodes represented the internal connections and genes, respectively. The number of edges from one node to another denoted the degree. Genes with higher degrees have a powerful ability to modulate other genes and were the core key genes in the signal-net.

### Co-expression network analysis (lncRNA–mRNA)

LncRNA–mRNA co-expression network analysis was conducted based on the normalized signal intensity of differentially expressed mRNAs and lncRNAs. The R function ‘cor.test’ (Hmisc and corrplot) was used to calculate the Pearson's correlations for each pair of mRNA-lncRNA. The mRNA–lncRNA pairs with Pearson’s correlations of > 0.9 were included to construct the visualization network using Cytoscape software (version 3.6.0). The degree was calculated to measure the centrality of a gene or lncRNA within a network.

### Real-time quantitative polymerase chain reaction (RT-qPCR)

Some of the core differentially expressed lncRNAs and mRNAs identified in microarray analysis were validated by RT-qPCR. The gene primers are listed in Additional file 1: Table S1. The housekeeping gene GAPDH was used as an internal control. We performed RT-qPCR using SYBR green reagent (Bio-Rad, Hercules, CA, USA) as per the manufacturer’s instructions and calculated the sample mean to ensure data stability. The relative expression levels of lncRNAs and mRNAs were calculated using the comparative CT method (2^−ΔΔCT^).

### Statistical analysis

The software programs used for statistical analysis were GraphPad Prism 7.0 (GraphPad, Inc., La Jolla, CA, USA) and SPSS version 22.0 (SPSS, Inc., Chicago, IL, USA). Expression levels of lncRNAs and mRNAs between patients with asthma and healthy controls were evaluated by Student’s *t*-tests. |FC|> 1.5 and *P* < 0.05 were considered as statistically significant. For GO and KEGG analysis, two-tailed Fisher’s exact tests were performed, and *P* < 0.05 were considered statistically significant. The experimental data for RT-qPCR analysis were represented as the median (interquartile range). Validation analysis was carried out using Mann–Whitney *U* test, and a *P* < 0.05 was considered statistically significant.

## Results

### Baseline characteristics

Ten patients with asthma and nine healthy controls participated in this study. Table 1 shows the baseline clinical and demographic characteristics of the participants in this study. No differences in age, sex, body mass index, pulse oximetry saturation, smoking history, FEV1, FEV_1_ as the percentage of the predicted value FEV_1_ to forced vital capacity, or blood eosinophils were observed between the two groups. Fractional exhaled nitric oxide and basophils percent were significantly higher in patients with asthma than in normal controls (*P* < 0.05). The asthma control test and asthma control questionnaire-7 scores of patients with asthma were 20 (19, 23) and 0.7 (0.6, 1.0), respectively.

**Table 1:** Baseline characteristics of the study participants

Characteristics	Asthma (n = 10)	Control (n = 9)
Age (years)	33.5 (28.8, 38.0)	35 (34, 38)
Gender (male, %)	50.0	55.5
Body mass index (BMI, kg/m^2^)	23.6 (21.6, 25.7)	23.7 (21.3, 25.8)
Pulse oximetry saturation (SpO_2_, %)	97.0 ( 96.0, 97.3)	99.0 (98.5, 99.0)***
Current/ex-smoker	1	0
Blood white blood cells count (cells/μL)	5300.0 (4700, 7400)	5770.0 (5130.0, 7370)
Blood eosinophils count (cells/μL)	150.0 (135, 220)	290 (180.0, 415)
Blood eosinophils (%)	4.0 (2.5, 4.5)	4.0 (3.2, 8.5)
Blood basophils (%)	0.0 (0.0, 0.2)	0.4 (0.4, 0.8)**
Blood neutrophils (%)	62.8 (58.6, 66.9)	59.0 (57.3, 60.9)
Blood lymphocytes (%)	28.6 (26.4, 35.6)	28.3 (26.9, 32.7)
Blood monocytes (%)	4.0 (2.5, 4.5)	5.2 (3.2, 5.7)
Forced expiratory volume in 1 s (FEV1, L)	3.1 (2.8, 3.6)	3.3 (2.7, 3.7)
FEV1% pred (%)	84.4 (74.8, 102.4)	96.0 (92.5, 103.5)
FEV1/forced vital capacity (FVC) (%)	84.4 (74.8, 102.4)	83.4 (77.7, 88.2)
Fractional exhaled nitric oxide (FeNO, bbp)	72.0 (23.0, 128.3)	12.0 (10.0, 29.0)*
Asthma control test (ACT) score	20 (19, 23)	NA
Asthma control questionnaire-7 (ACQ-7) score	0.7 (0.6, 1.0)	NA
Medication status		
Short-acting beta-2 agonist (SABA)	3	0
Long-acting beta-2 agonist (LABA)	0	0
Long-acting muscarinic antagonist (LAMA)	0	0
LABA + inhaled corticosteroid (ICS)	1	0
LAMA + ICS	7	0
Theophylline	1	0

Data are presented as the median (interquartile range) unless otherwise stated

*P < 0.05; **P < 0.01 ***P < 0.001

### Identification and differential expression of mRNAs and lncRNAs in patients with asthma versus healthy controls

**Fig. 1:**
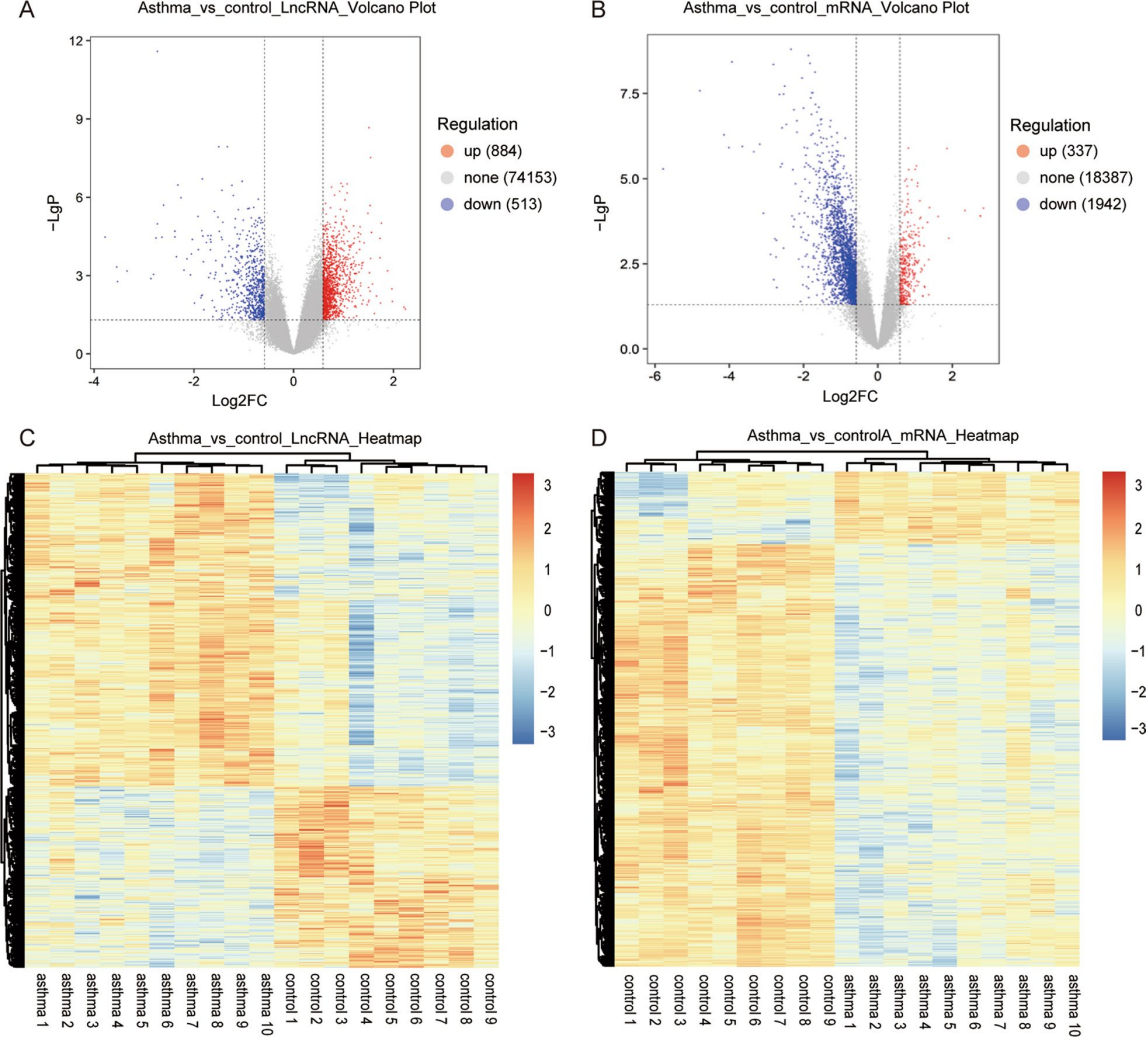
Differential expression of lncRNAs and mRNAs in patients with asthma. Volcano plots are used to distinguish between differentially expressed lncRNAs (**A**) and mRNAs (**B**). Red and blue indicate up- and down-regulation, respectively. Hierarchical clustering analysis of dysregulated lncRNAs (**C**) and mRNAs (**D**). Relative lncRNA or mRNA expression is depicted according to the color scale. Red indicates elevated expression and blue indicates reduced expression

Microarray analysis of the whole transcriptome was performed to explore the differential expression of mRNAs and lncRNAs between patients with bronchial asthma and healthy controls. The cutoff criteria of statistical significance were as mentioned above (|FC|> 1.5 and *P* < 0.05). Microarray analysis detected 75,550 lncRNAs in the PBMC samples, among which 1,397 lncRNAs (884 upregulated and 513 downregulated lncRNAs) showed significant differences (Fig. 1A). A total of 20,666 mRNAs was detected, including 337 upregulated and 1942 downregulated mRNAs between patients with asthma and healthy controls (Fig. 1B). Hierarchical clustering of lncRNAs and mRNAs was performed to distinguish patients with asthma from healthy controls using Pearson’s correlation (Fig. 1C,D). The top-ten most significant lncRNAs and mRNAs are shown in Additional file 1: Tables S2 and S3.

### Functional annotation and pathway analysis

**Fig. 2:**
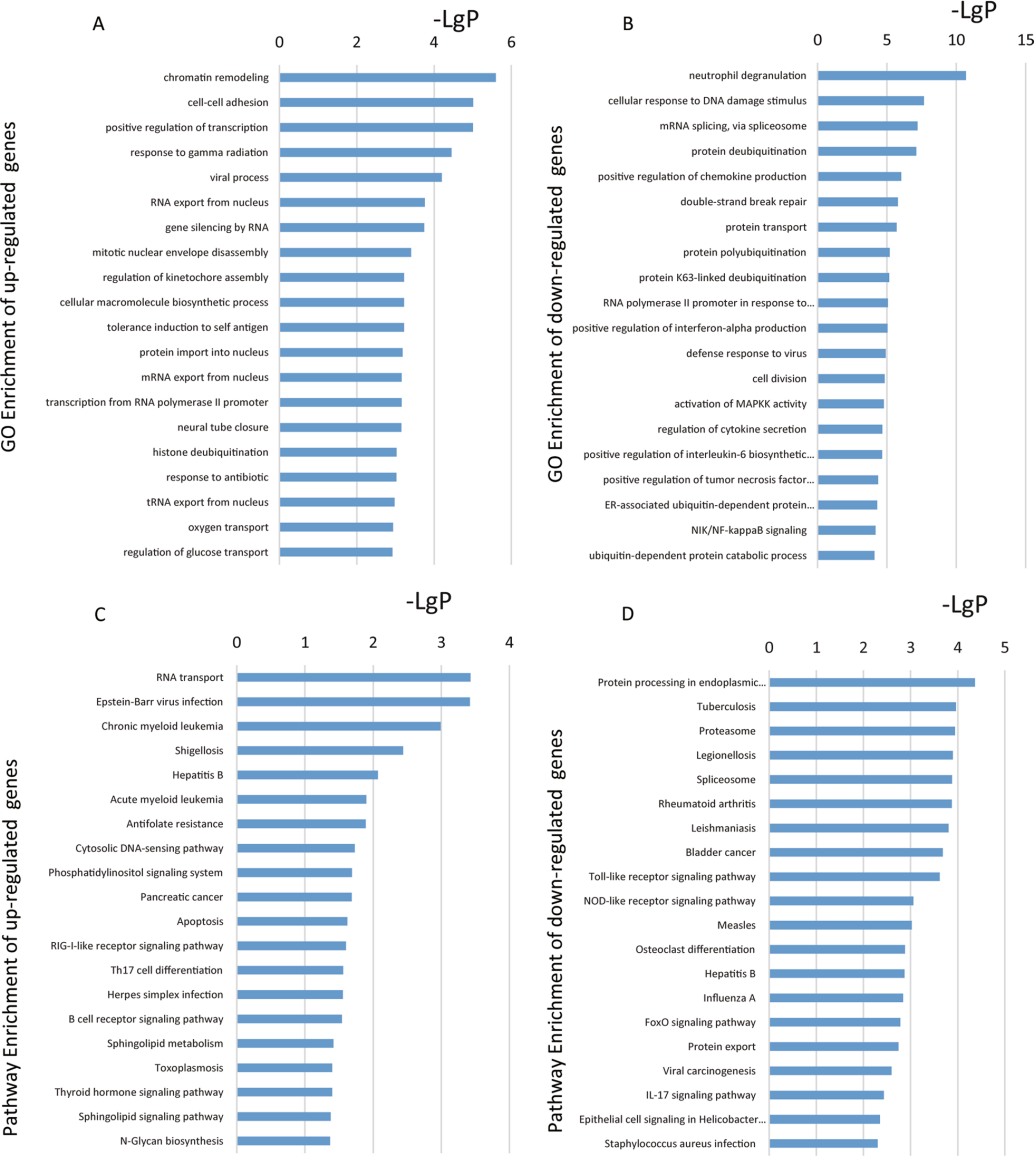
GO and KEGG pathway analyses. GO analysis of significantly upregulated (**A**) and downregulated (**B**) mRNAs clustered in the BP. KEGG pathways analysis of differentially upregulated (**C**) and downregulated mRNAs (**D**). The abscissa shows ‐LogP, and the ordinate shows GO terms or KEGG pathways. GO, Gene Ontology; KEGG, Kyoto Encyclopedia of Genes and Genomes; BP, biological process

To further analyze the differentially expressed mRNA, we performed KEGG pathway and GO analyses. Upregulated GO functions included chromatin remodeling, cell–cell adhesion, positive regulation of transcription, response to gamma radiation, viral process, RNA export from the nucleus, and gene silencing via RNA (Fig. 2A). Downregulated GO functions included neutrophil degranulation, cellular response to DNA damage stimuli, mRNA splicing, protein deubiquitination, positive regulation of chemokine production, and double-strand break repair (Fig. 2B). In KEGG pathway analysis, the pathways enrichment of up-regulated genes included RNA transport, apoptosis, the retinoic acid-inducible gene I (RIG-I)-like receptor signaling pathway, and T helper 17 cell differentiation (Fig. 2C) [16]. The pathways enrichment of down-regulated genes included the Toll-like receptor (TLR) signaling pathway, nucleotide-binding oligomerization domain (NOD)-like receptor signaling pathway, Forkhead Box class O (FOXO) signaling pathway, and interleukin signaling pathway (Fig. 2D) [16].

### Global signal transduction network (signal-net)

**Fig. 3:**
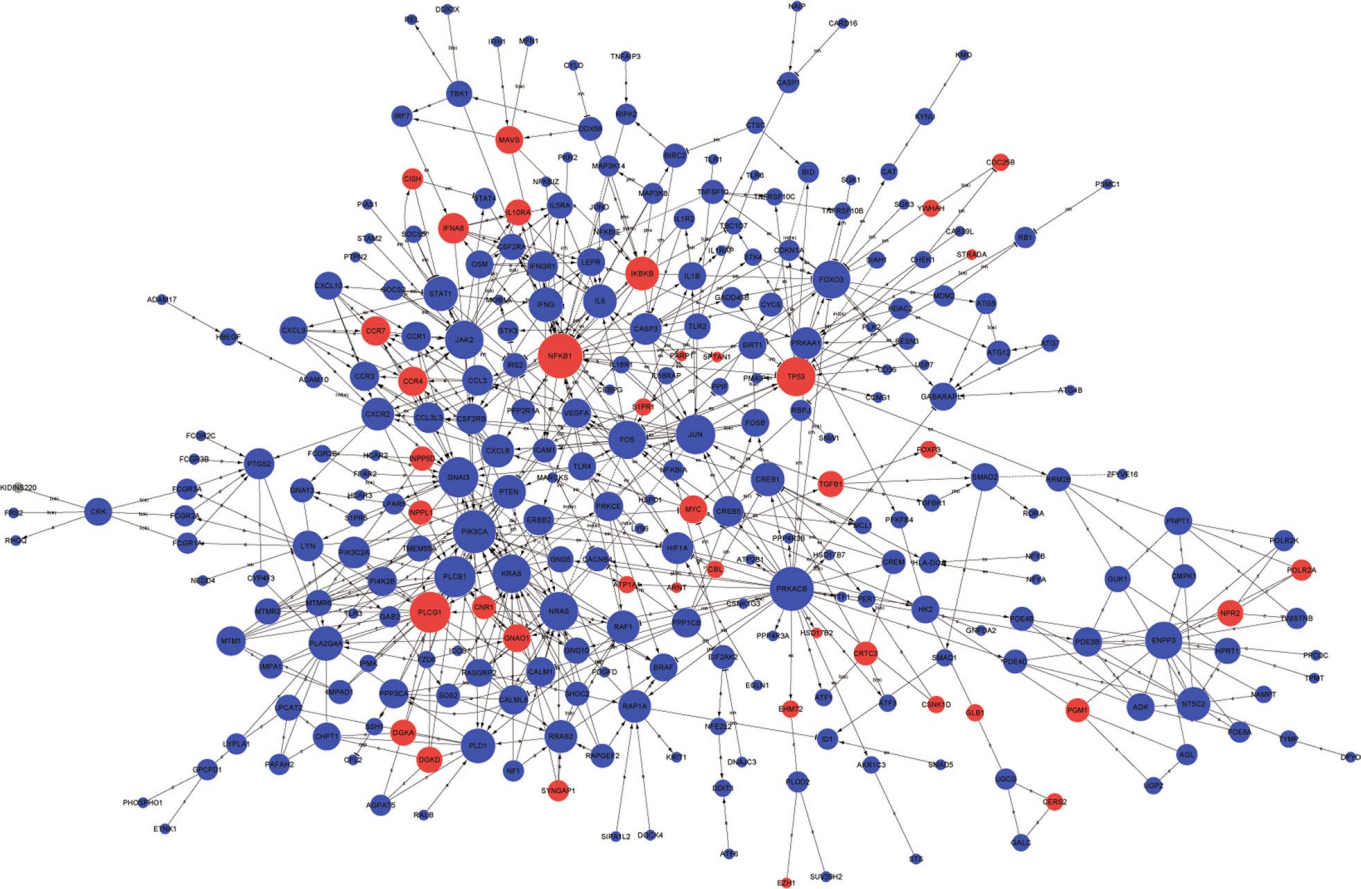
Global signal transduction network analysis of asthma mRNAs. In the constructed network, nodes represent mRNAs, the size of the node’s area represents the value of the degree, and red indicates up-regulation and blue indicates down-regulation. The nodes are connected by an edge. The acronyms a, b, c, p, inh, and ind (e) indicate activation, binding, compound, phosphorylation, inhibition, and indirect effect, respectively

A global signal transduction network was constructed to identify the core key genes and mRNAs (Fig. 3). *NFKB1* showed the highest degree value and centrality range, reflecting its centrality in the network as it connected with the most genes. In addition, *PRKACB*, *PIK3CA*, *PLCB1*, *PLCG1*, *PLCG1*, *GNAI3*, *JUN*, *P53*, *KRAS*, *NRAS*, *JAK2*, *FOXO3*, and a few other genes were determined to have important functions in the signal-net with high degree values (Table 2).

**Table 2:** Core mRNAs selected using the gene–gene functional interaction network

Gene symbol	Style	Betweenness centrality	Degree
*NFKB1*	Up	0.052151	35
*PRKACB*	Down	0	32
*PIK3CA*	Down	0.033763	29
*PLCB1*	Down	0.04064	24
*PLCG1*	Up	0.02381	23
*GNAI3*	Down	0.0408	21
*JUN*	Down	0.030527	20
*TP53*	Up	0.040001	19
*KRAS*	Down	0.026864	18
*NRAS*	Down	0.026311	18
*JAK2*	Down	0.011193	18
*FOXO3*	Down	0.024722	17
*FOS*	Down	0.019986	17
*ENPP3*	Down	0.014337	16
*STAT1*	Down	0.014575	12
*IFNG*	Down	0.013518	12
*PLA2G4A*	Down	0.006587	12
*PLD1*	Down	0.005553	12
*CREB1*	Down	0.006042	11
*IL6*	Down	0.00833	11

Betweenness centrality ≥ 0.005. Betweenness centrality is an indicator of a gene’s centrality in a network. It is equal to the number of shortest paths from all vertices to all others that pass through that gene. The degree of a gene was defined as the number of directly linked genes within a network

### LncRNA–mRNA co-expression network

The 519 mRNAs and 380 lncRNAs showed a co-expression relationship. The lncRNA–mRNA co-expression network was mainly used to identify relationships between these mRNAs and lncRNAs, comprising 899 nodes and 1146 edges (Additional file 1: Figure S1). We focused on the core mRNAs with high degrees in signal-net and highly expressed mRNAs with high FCs in microarray analysis. We found that lncRNA KB-67B5.12 was negatively correlated with *PIK3CA*, whereas lncRNA MTCYBP3 and lncRNA AC019050.1 were positively correlated with *JUN* and *FOXO3*, respectively. Interleukin-8/C-X-C motif chemokine ligand 8 (*CXCL8*) and *G0*/*G1* switch gene 2 (*G0S2*) showed the largest difference in FC. LncRNA NONHSAT115963 was positively corelated with *CXCL8* and NONHSAT122646 was negatively correlated with *G0S2* (Table 3).

**Table 3:** Correlation between lncRNAs and core mRNAs

mRNA	lncRNA	Pearson’s correlation	Regulation
*CXCL8*	NONHSAT115963	0.917**	Positive
*FOXO3*	AC019050.1	− 0.925**	Negative
*JUN*	MTCYBP3	0.925**	Positive
*PIK3CA*	KB-67B5.12	0.935**	Positive
*G0S2*	HNRNPA1P12	− 0.901**	Negative

**P < 0.01

### RT-qPCR validation

**Fig. 4:**
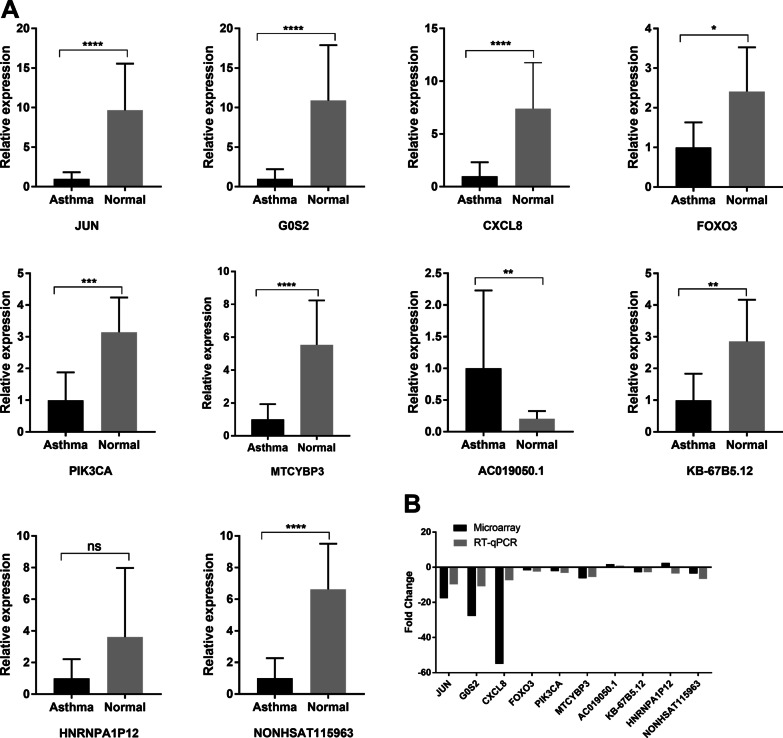
RT‐qPCR validation of selected mRNAs and lncRNAs. **A** Relative expression levels of the mRNAs and lncRNAs between the patients with asthma and healthy control assessed by RT-qPCR. The y-axis represents the relative expression (2^−ΔΔCT^). Data are presented as the median (interquartile range). All data were normalized to *GAPDH* expression. **B** Comparison of mean fold-changes between microarray data and RT-qPCR results. **P < 0.01; ***P < 0.001; **** *P* < 0.0001; ns, no significance. RT-qPCR, real-time quantitative polymerase chain reaction

We conducted RT-qPCR to verify the stability and reliability of the microarray analysis. Five core mRNAs and their related lncRNAs were selected for RT-qPCR (Fig. 4). The results of RT-qPCR analysis of nine genes were consistent with the microarray results. AC019050.1 was upregulated, whereas *JUN*, *GOS2*, *CXCL8*, *FOXO3*, *PIK3CA*, *MTCYBP3*, *KB-67b5.12*, and *NONHSAT115963* were downregulated. Only the lncRNA of *HNRNPA1P12* showed an opposite trend between RT-qPCR and microarray analysis.

## Discussion

Asthma is a common health issue which poses an economic and social burden on patients. However, the pathogenesis of this disease remains poorly understood. Recent studies have highlighted the potential roles of lncRNAs in the pathogenesis of asthma. Lin reported that the lncRNA TUG1 promoted airway smooth muscle cell proliferation and migration, contributing to asthma [17]. The lncRNA ANRIL/miR-125a axis may be related to inflammation, exacerbation, and the severity of bronchial asthma [18]. In this study, we give a comprehensive analysis of the various mRNA and lncRNAs and their interaction in asthma and healthy controls. 884 upregulated and 513 downregulated lncRNAs were identified between ten patients with asthma and nine healthy controls. Most of the differentially expressed genes were involved in the pathways associated with inflammation and cell survival, and these factors may serve as biomarkers for bronchial asthma.

GO and KEGG pathway analyses were performed to identify enriched biological functions. According to the results of the GO analysis, DEGs were involved in processes such as cell–cell adhesion, neutrophil degranulation, cellular response to DNA damage stimuli, and positive regulation of chemokine production. Previous studies demonstrated that these are important biological processes in asthma. Dysregulation of cell–cell adhesion leads to epithelial barrier destruction, which may facilitate penetration of environmental allergens, thereby activating the innate immune responses and increasing asthma severity and susceptibility [19]. Airway neutrophilia is associated with asthma severity and is refractory to treatment with corticosteroids. Neutrophils can kill pathogens but may also damage the airways by affecting proteases and reactive oxygen species [20]. Airway inflammation, oxidative damage, DNA damage, and repair protein levels in asthma have been found to be increased [21]. Asthma is known as a chronic inflammatory disease, and processes related to chemokine production, MAPK activation, and NF-κB activation were detected in this study.

KEGG pathway analysis revealed that the differentially expressed mRNAs are mainly related to apoptosis and *TLR*, RIG-I-like receptor, NOD-like receptor, *FOXO,* and T helper 17 signaling pathway. Some of these pathways have been demonstrated crucial to the onset of asthma. For example, CD9+ B cells induce effector T cell cycle arrest in sub G0/G1 and apoptosis in asthmatic mice [22]. RIG-I has long been known to serve as a pattern recognition receptor for viral detection. However, this receptor was also found to serve several functions as an RNA-responsiveness protein for various cellular activities, including cell development and proliferation [23]. However, the role of RIG-I in asthma requires further study. TLRs recognize microbial, endogenous molecules, and environmental allergens and play an immune-modulatory role in asthma development [24]. Bacterial infection-mediated activation of NOD-like receptors triggers allergic asthma by activating eosinophils, which interact with bronchial epithelial cells in the airway [25]. *FOXO* has multiple biological functions, such as modulation of embryonic endothelial stem cell survival [26], regulation of ischemic brain injury [27], and vascular disease [28], which is a newly investigated aspect of asthma. In this study, we identified some classical biological processes and new processes in asthma, providing a foundation for further study.

We constructed a global signal transduction network to identify core genes with crucial roles in the pathogenesis of asthma. The mRNAs *NFKB1*, *PRKACB*, *PIK3CA*, *PLCB1*, and *PLCG1* had the top five degrees in the gene signal network. *NFKB1* is the NF-κB p105 subunit, a typical inflammation pathway gene, and is associated with asthma. The protein kinase cAMP-dependent catalytic subunit β (*PRKACB*) is a member of the serine/threonine protein kinase family. *PRKACB* plays a key role in apoptosis and cell differentiation and proliferation [29]**.** The phosphatidylinositol-4,5-bisphosphate 3-kinase catalytic subunit alpha (*PIK3CA*) gene encodes the PI3K-p110α protein, which activates the PI3K pathway and leads to dysregulated cell proliferation [30]**.** *PLCB1* encodes the protein phospholipase C *β*1 and plays an important role in intracellular transduction of many extracellular signals [31]. The main function of *PLCG1* is to encode phospholipase C γ1, which mainly catalyzes the hydrolysis of phosphatidylinositol 4,5‐bisphosphate to generate second messenger molecules [32]. Most of these genes have not been sufficiently studied in asthma and require further investigation. However, some mRNAs showing high degrees were not correlated with lncRNAs.

LncRNAs exert their functions by modulating mRNA processing and post-transcriptional regulation. Based on the lncRNA–mRNA co-expression network, gene signal network, and FC in the based expression list, we identified the following lncRNAs: AC019050.1, MTCYBP3, KB-67B5.12, NONHSAT122646, HNRNPA1P12, and their related mRNAs (*FOXO3*, *JUN*, *PIK3CA*, *CXCL8*, and *G0S2*) as key factors. FOXO is a subfamily of forkhead transcription factors and plays key roles in regulating many pathways that regulate processes such as apoptosis, insulin signaling, DNA repair, oxidative stress resistance, and longevity [33, 34]. As the core component of the AP-1 transcription factor complex, JUN is an important factor in cell survival, cell proliferation, and movement [35]. According to previous studies, c-Jun can be a therapeutic target for cancer, vascular remodeling, acute inflammation, and rheumatoid arthritis [36]. There is no clear conclusion regarding the functions of G0S2 in asthma. G0S2 is highly expressed in the liver, heart, and skeletal muscle [37, 38] and is expressed at low levels in some tumors [39]. The roles of these lncRNAs and related mRNAs in asthma require further analysis.

Our study had some limitations. First, there were too few patients enrolled in this study and the conclusion was easily influenced by the heterogenicity of sample. And the clinical data was not sufficient to identify the phenotypes of asthma. The results need to be validated in a bigger cohort of patients and in different asthma phenotypes in the future. Second, the patients were assessed during a stable period of asthma, and some patients took medications such as ICS treatment, which may have altered their gene expression. Third, the functions of the differentially expressed mRNAs and lncRNAs were based on bioinformatics analysis, and therefore must be verified in animal experiments. Finally, there may be differences in the composition of PBMCs between the healthy control and asthma groups which may lead to the detection of different genes. If single-cell sequencing technology can be used to detect gene expression in different components of cells, the results may be more accurate; this will be the focus of our future study.

## Conclusion

Using microarray analysis, we identified core mRNAs and their related lncRNAs, as well as predicted the possible altered biological processes and signaling pathways associated with asthma. This study provides insights into the investigated targets of asthma, which may be useful as lncRNA-mediated therapy for asthma.

## Supplementary Information

**Additional file 1. Supplementary Figure 1**. LncRNA-mRNA expression network. **Supplementary Table 1**. Primers used for real time-polymerase quantitative chain reaction. **Supplementary Table 2**. Ten most up- and down-regulated mRNAs in patients with asthma compared to in normal controls. **Supplementary Table 3**. Ten most up- and down-regulated lncRNAs in patients with asthma compared to in normal controls.

## Data Availability

The datasets used or analyzed during the current study were deposited at NCBI, under accession number GSE165934 (available at: https://www.ncbi.nlm.nih.gov/bioproject/?term=GSE165934).

## Abbreviations

CXCL8: C-X-C motif chemokine ligand 8
FOXO: Forkhead box class O
FEV1: Forced expiratory volume in first second
G0S2: G0/G1 switch gene 2
GO: Gene ontology
Th: T helper T cell
KEGG: Kyoto encyclopedia of genes and genomes
LncRNA: Long noncoding RNA
NOD: Nucleotide-binding oligomerization domain
PBMCs: Peripheral blood mononuclear cells
RT-qPCR: Real-time polymerase quantitative chain reaction
RIG-I: Retinoic acid-inducible gene I
TLR: Toll-like receptor
