# Supplementary material for: Integrated analysis of mRNA and long noncoding RNA profiles in peripheral blood mononuclear cells of patients with bronchial asthma
Source: BMC Pulm Med. 2022 May 2;22:174. doi: 10.1186/s12890-022-01945-9 (PMC9059365; doi:10.1186/s12890-022-01945-9)
Supplement: Supplementary file 1 — Additional file 1. Supplementary Figure 1. LncRNA-mRNA expression network. Supplementary Table 1. Primers used for real time-polymerase quantitative chain reaction. Supplementary Table 2. Ten most up- and down-regulated mRNAs in patients with asthma compared to in normal controls. Supplementary Table 3. Ten most up- and down-regulated lncRNAs in patients with asthma compared to in normal controls. [file 12890_2022_1945_MOESM1_ESM.docx]

Supplementary Material


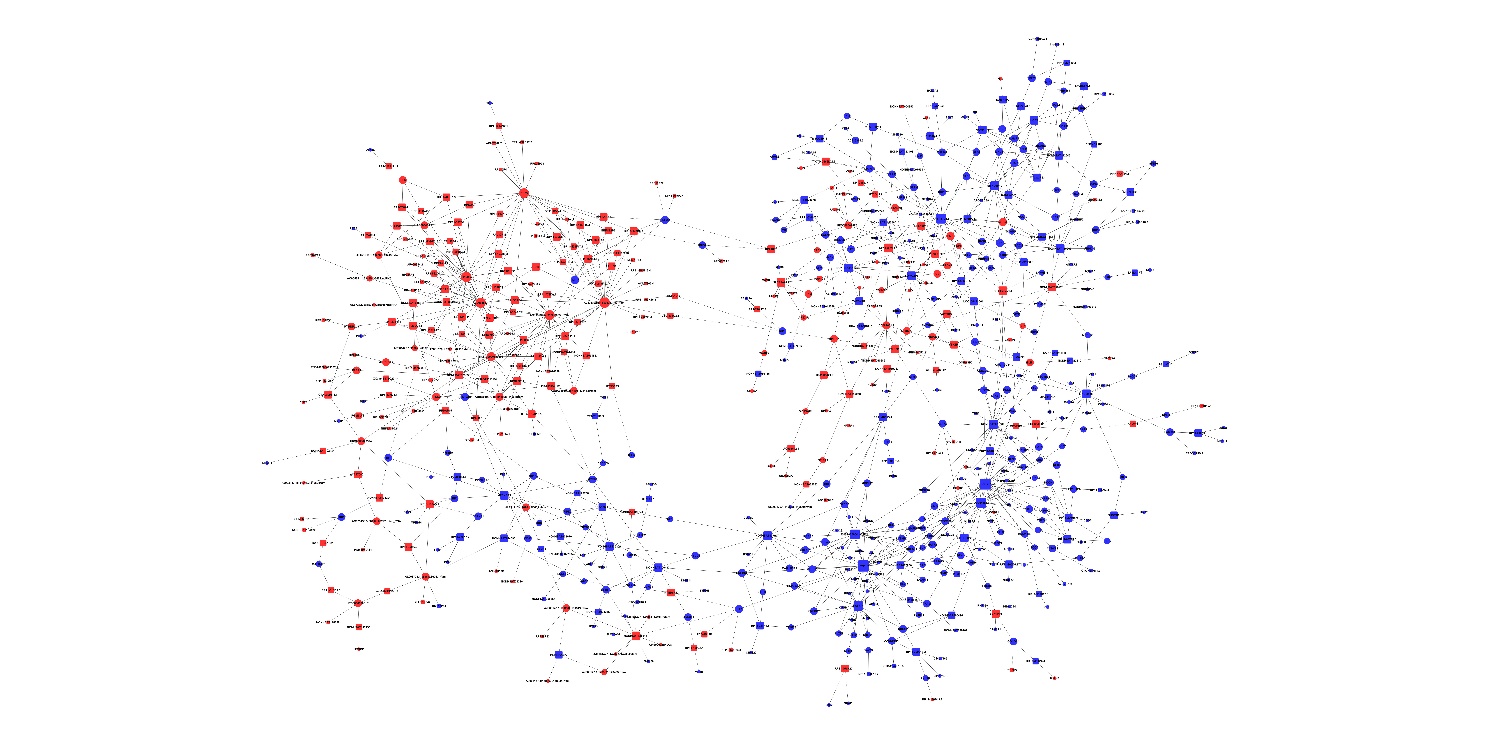


**Supplementary Figure 1.** **LncRNA-mRNA expression network.** This network consisted of a coexpression relationship between lncRNAs and mRNAs. Blue represents downregulation and red represents upregulation. Circle nodes represent mRNAs and box nodes represent lncRNAs. The node degree is indicated by the size. An edge represents a coexpression relationship between an mRNA and lncRNA.

**Supplementary Table 1. Primers used for real time-polymerase quantitative chain reaction**

| **Gene symbol** | **Forward primer (5′-3′)** | **Reverse primer (5′-3′)** |
| --- | --- | --- |
| *CXCL8* | ACTCCAAACCTTTCCACCCC | ATGAATTCTCAGCCCTCTTCAA |
| *FOXO3* | CGGACAAACGGCTCACTCT | GGACCCGCATGAATCGACTAT |
| *G0S2* | CCAAGGAGATGATGGCCCAG | GCTGCACACAGTCTCCATCA |
| *JUN* | TCCAAGTGCCGAAAAAGGAAG | CGAGTTCTGAGCTTTCAAGGT |
| *PIK3CA* | CCACGACCATCATCAGGTGAA | CCTCACGGAGGCATTCTAAAGT |
| *MTCYBP23* | GCCAACCCCCTCAATACCTC | GGGCCAGTACACCTCCTAGT |
| *NONHSAT115963* | CAATGAGGGGCAGAGAATTATGG | AAAAGAACCTGCTTTTGTTGACTGA |
| *AC019050.1* | ACGGCCAAAACTTCAACACC | GTGGAAGGACCGAGCATACA |
| *HNRNPA1P12* | GGTGCCCACTTAACTGTGAAAAA | TTGCTGTGACGGGGCT |
| *KB-67B5.12* | TTGTTTATGTTTGGCTGGAAGAGTT | AAGTTGAGAAGCTCATCACTGGT |

**Supplementary Table 2. Ten most up- and down-regulated mRNAs in patients with asthma compared to in normal controls**

| **Gene Symbol** | **FC** | **Regulation** | **P Value** | **Chromosome** |
| --- | --- | --- | --- | --- |
| *CXCL8* | -54.948 | down | 5.23627E-06 | chr4 |
| *G0S2* | -27.665 | down | 2.60256E-08 | chr1 |
| *JUN* | -17.63 | down | 5.16639E-07 | chr1 |
| *FOS* | -16.111 | down | 1.24046E-06 | chr14 |
| *CD69* | -15.242 | down | 3.73663E-09 | chr12 |
| *IL1B* | -12.553 | down | 1.14173E-06 | chr2 |
| *RGS1* | -10.126 | down | 1.63882E-06 | chr1 |
| *DUSP1* | -9.063 | down | 9.77696E-07 | chr5 |
| *NAMPT* | -8.456 | down | 0.000105139 | chr7 |
| *IFIT1* | -7.413 | down | 0.001680308 | chr10 |
| *ATP8B2* | 2.621 | up | 7.14415E-05 | chr1 |
| *IGLL5* | 2.585 | up | 0.025674158 | chr22 |
| *EMD* | 2.514 | up | 0.000191305 | chrX |
| *CCR7* | 2.395 | up | 0.015484847 | chr17 |
| *CCR4* | 2.378 | up | 0.003516618 | chr3 |
| *S1PR1* | 2.362 | up | 0.000949181 | chr1 |
| *HBA2* | 2.313 | up | 0.02824217 | chr16 |
| *HBA1* | 2.297 | up | 0.028026586 | chr16 |
| *RPL27* | 2.282 | up | 0.00480925 | chr17 |
| *SYNGAP1* | 2.25 | up | 0.004347276 | chr6 |

FC: fold-change

**Supplementary Table 3. Ten most up- and down-regulated lncRNAs in patients with asthma compared to in normal controls**

| **Gene Symbol** | **FC** | **Regulation** | **P Value** | **Chromosome** |
| --- | --- | --- | --- | --- |
| *NONHSAT122646* | -13.737 | down | 3.37897E-05 | chr7 |
| *RP11-22N19.2* | -6.635 | down | 1.04134E-05 | chr7 |
| *MIR222HG* | -6.635 | down | 2.6107E-12 | chrX |
| *NONHSAT079756* | -5.205 | down | 2.0095E-05 | chr20 |
| *RP6-99M1.3* | -4.993 | down | 3.42689E-07 | chrX |
| *LUCAT1* | -4.595 | down | 0.000628063 | chr5 |
| *NONHSAT122608* | -4.408 | down | 0.000230339 | chr7 |
| *RP11-300J18.2* | -4.141 | down | 0.000149366 | chr14 |
| *NONHSAT122736* | -4.084 | down | 4.14781E-05 | chr7 |
| *NONHSAT123408* | -3.784 | down | 5.44169E-06 | chr7 |
| *NONHSAT039609* | 4.724 | up | 0.019442522 | chr14 |
| *NONHSAT136020* | 3.945 | up | 0.009910918 | chr14 |
| *LOC100506159* | 3.681 | up | 0.000663292 | chr12 |
| *NONHSAT136018* | 3.34 | up | 0.01141111 | chr22 |
| *NONHSAT102434* | 3.16 | up | 0.006133622 | chr5 |
| *lnc-EPHA6-1:1* | 2.99 | up | 0.002100903 | chr3 |
| *NONHSAT081067* | 2.949 | up | 2.22742E-05 | chr21 |
| *NONHSAT136019* | 2.949 | up | 2.22742E-05 | chr22 |
| *RP4-539M6.21* | 2.868 | up | 0.000781358 | chr22 |
| *NONHSAT136004* | 2.848 | up | 0.008838896 | chr22 |

FC: fold-change
